# Centenarians consistently present a younger epigenetic age than their chronological age with four epigenetic clocks based on a small number of CpG sites

**DOI:** 10.18632/aging.204316

**Published:** 2022-10-03

**Authors:** Antoine Daunay, Lise M. Hardy, Yosra Bouyacoub, Mourad Sahbatou, Mathilde Touvier, Hélène Blanché, Jean-François Deleuze, Alexandre How-Kit

**Affiliations:** 1 Laboratory for Genomics, Foundation Jean Dausset – CEPH, Paris, France; 2 Laboratory of Excellence GenMed, Paris, France; 3 Sorbonne Paris Nord University, Nutritional Epidemiology Research Team (EREN), Epidemiology and Statistics Research Center Inserm U1153, Inrae U1125, Cnam, University of Paris (CRESS), Bobigny, France; 4 Centre de Ressources Biologiques, CEPH Biobank, Foundation Jean Dausset – CEPH, Paris, France; 5 Centre National de Recherche en Génomique Humaine, CEA, Institut François Jacob, Evry, France

**Keywords:** epigenetic clock, DNAmage, centenarians, DNA methylation, pyrosequencing, longevity

## Abstract

Aging is a progressive time-dependent biological process affecting differentially individuals, who can sometimes present exceptional longevity. Epigenetic alterations are one of the hallmarks of aging, which comprise the epigenetic drift and clock at DNA methylation level. In the present study, we estimated the DNA methylation-based age (DNAmage) using four epigenetic clocks based on a small number of CpGs in French centenarians and semi-supercentenarians (CSSC, n=214) as well as nonagenarians' and centenarians' offspring (NCO, n=143) compared to individuals from the French general population (CG, n=149). DNA methylation analysis of the nine CpGs included in the epigenetic clocks showed high correlation with chronological age (-0.66>R>0.54) and also the presence of an epigenetic drift for four CpGs that was only visible in CSSC. DNAmage analysis showed that CSSC and to a lesser extend NCO present a younger DNAmage than their chronological age (15-28.5 years for CSSC, 4.4-11.5 years for NCO and 4.2-8.2 years for CG), which were strongly significant in CSSC compared to CG (p-values<2.2e-16). These differences suggest that epigenetic aging and potentially biological aging are slowed in exceptionally long-lived individuals and that epigenetic clocks based on a small number of CpGs are sufficient to reveal alterations of the global epigenetic clock.

## INTRODUCTION

Aging is a natural time-dependent biological process occurring until death in most living organisms including humans, which is characterized by the progressive decline of overall fitness as well as several molecular, cellular and physiological functions, under the influence of genetic, environmental and stochastic factors [1–3]. Among molecular hallmarks of aging, epigenetic modifications, including notably histone modifications, DNA methylation and chromatin remodeling, have been widely studied and described in mammals and humans and proposed as potential factors accompanying and even causing the aging process [1, 4, 5].

In this context, the study of DNA methylation in human aging has revealed the occurrence of two types of age-related DNA methylation changes [6]. The first, known as epigenetic drift, is characterized by the progressive divergence of the methylome of individuals acquired environmentally and stochastically across their lifespan [7, 8], which even affects monozygotic twins [9, 10]. The second type of DNA methylation changes is called the epigenetic clock and refers to all age-related DNA methylation variations that consistently increase or decrease in every individual, thereby correlating to their chronological age [6, 11].

The latter type of epigenetic modifications has been widely used as biomarkers of aging in several age-prediction models to estimate the chronological and biological age of individuals, mainly from blood DNA samples [6, 11, 12]. These models are based on multiple regression, machine learning and deep learning approaches using either a large number of CpGs requiring high-throughput technologies such as genome-wide epigenotyping array or a smaller number of CpGs requiring high resolution locus-specific methods such as pyrosequencing [7, 13–18]. DNA methylation-based age (DNAmage) prediction has proven to be of great interest in several bio-medical applications. It could notably give a better estimation of the biological age than chronological age [19, 20] and could also be a good indicator or predicator of different risks, health conditions and age-related diseases when compared to the chronological age [21–26].

Long-lived individuals (LLI) are defined as individuals over 90 years old greatly surpassing the human life expectancy and are also considered as an appropriate model for healthy aging studies due to their greater healthspan [27–29]. To date, only three studies have evaluated four epigenetic clocks based on epigenotyping arrays using a large number of CpG loci (Hanum clock=71 CpGs, Horvath clock=353 CpGs, Levine clock=513 GpGs and Lu clock=184 CpGs) in Italian, Australian and Israeli LLI, including nonagenarian, centenarians and/or semi-supercentenarians, as well as their offspring [30–32]. The results showed that these LLI and their offspring presented a younger DNAmage than their chronological age, suggesting slower aging rates in these groups of individuals [30–32].

**Figure 1:**
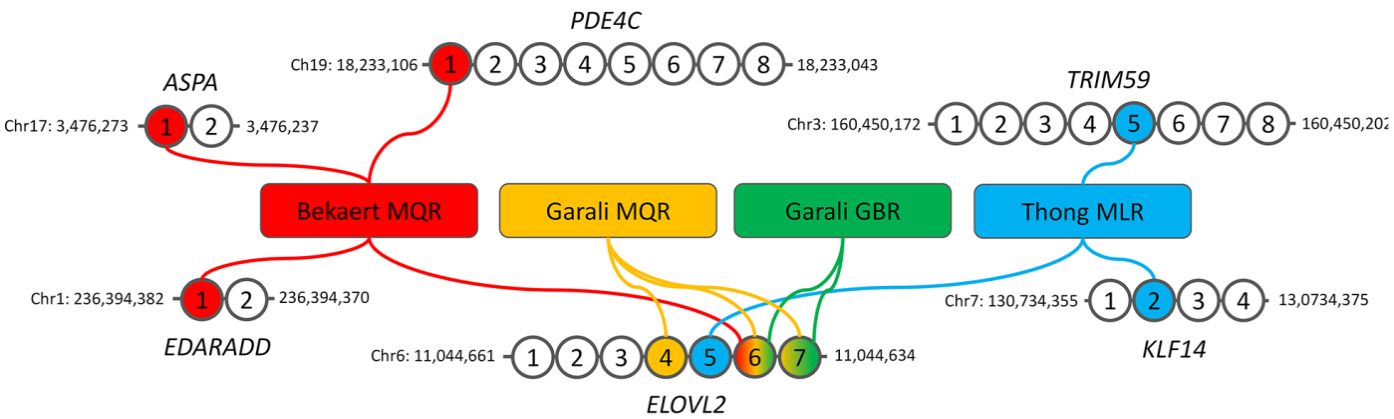
**Description of the four DNA methylation-based epigenetic clocks used in our study.** The genomic location of the first and last CpGs analyzed by pyrosequencing are given for each gene. MQR: multiple quadratic regression model, GBR: machine-learning gradient boosting regressor model, MLR: multiple linear regression model.

In the present study, we investigated the DNAmage of French LLI including centenarians and semi-supercentenarians (n=214), as well as nonagenarian’s and centenarian’s offspring (n=143) of the CEPH aging cohort [33, 34] using blood extracted DNA and four epigenetic clocks based on a small number of CpGs and locus-specific pyrosequencing [17]. These clocks, known as Bekaert, Thong, Garali MQR and Garali GBR clocks, were developed from 2 to 4 CpGs located in the promoters of 1 to 4 genes (*ASPA*, *EDARADD*, *ELOVL2*, *KLF14*, *PDE4C* and *TRIM59*) using multiple linear or quadratic regressions, and machine learning gradient boosting regressor (Figure 1) [17, 18, 35, 36]. The obtained DNAmage were compared to a control group composed of individuals (n=149) from the French general population from the SU.VI.MAX cohorts and EFS (French Blood Establishment) [33, 37]. Our study is the first to evaluate DNAmage in such a large cohort of centenarians using four epigenetic clocks based on a small number of CpGs.

## MATERIALS AND METHODS

### Study participants

### The CEPH aging cohort

The CEPH Aging cohort was recruited during the years 1990 to 2000 in order to identify genetic factors associated to longevity in the French population. The cohort included 1561 French nonagenarians, centenarians, semi-supercentenarians and supercentenarians born between 1875 and 1910, as well as 468 of their offspring being part of 147 families [33, 34, 38]. 214 unrelated French centenarians and semi-supercentenarians and 143 nonagenarians’ and centenarians’ offspring were included in our study (Table 1).

**Table 1:** Descriptive statistics of the DNA samples used in our study.

**Cohort characteristics**	**Control groups of individuals from the French general population (n=149)**			**CEPH aging cohort (n=357)**	
	**SU.VI.MAX (n=118)**	**EFS (n=31)**	**All (n=149)**	**NCO (n=143)**	**CSSC (n=214)**
Age at collection (years), M ± SD (range)	55.3 ± 4.8 (38-61)	58.8 ± 3.2 (52-65)	56 ± 4.7 (38-65)	61.2 ± 6.1 (38-68)	101.3 ± 1.4 (100-107)
Age at death (years), M ± SD (range)	-	-	-	-	103 ± 2 (100-108)
Age at death of the oldest parent (years), M ± SD (range)	-	-	-	96.8 ± 3.1 (90-108)	-
Females, n (%)	52 (44.1%)	5 (16.1%)	57 (38.3%)	84 (58.7%)	181 (84.6%)

### Individuals from the SU.VI.MAX cohort and EFS

The control group was formed of French individuals from the SU.VI.MAX cohort and EFS (Table 1). The SU.VI.MAX study initially included 13 017 disease-free participants from the French general population, who were recruited in the 1990s in order to measure the health effects of antioxidants vitamins and minerals [37]. 118 individuals from the SU.VI.MAX cohort were included in our study (Table 1). Moreover, 31 self-reported healthy donors from the French blood bank were also included in our study (Table 1).

### DNA extraction and quantification

All DNA samples were extracted from the buffy coats isolated from blood samples of the participants and were provided by the CEPH and CNRGH Biobanks. DNA samples from all collections were quantified using Quant-IT™ dsDNA Broad-Range assay kit on a Synergy HTX (BioTek) or Qubit™ dsDNA BR assay Kit on a Qubit 3 Fluorometer (Thermo Fischer Scientific), according to the manufacturer’s instructions.

### DNA methylation analysis

To limit experimental bias that could arise during experiments, DNA samples from the different groups were included in each 96-well bisulfite-treated plate as well as a same commercial whole blood DNA sample (Promega). 500 ng of blood extracted DNA was bisulfite-treated using the EpiTect Bisulfite 96 Kit (Qiagen) according to the manufacturer’s instructions. 20 ng of bisulfite-treated DNA was used as template for each PCR reaction using six bisulfite-specific PCR primer pairs (*ASPA*, *EDARADD*, *ELOVL2*, *KLF14*, *PDE4C* and *TRIM59*) according to the PCR reaction and cycling conditions described in previous studies [17, 18]. After PCR, 10 μl of amplified product was purified and prepared for pyrosequencing according to the detailed protocol described previously [39, 40]. DNA methylation analysis was performed on a PyroMark Q96 MD using the PyroMark Gold SQA Q96 Kit (Qiagen) using the pyrosequencing primers and assays described in Daunay et al., [17] and the data were generated and analyzed with PyroMark CpG software (Qiagen). DNA methylation of the promega control DNA sample showed close values for each CpG sites between replicate experiments indicating no or very little technical variations due to batch effect (Supplementary Figure 1).

### Epigenetic clocks and age predictions

Four blood-based DNA methylation-based age predictions models were used including Bekaert clock [35], Thong clock [36], Garali MQR and Garali GBR clocks [18]. Bekaert’s clock is based on a multiple quadratic regression model (MQR) using 4 CpGs located in *ASPA*, *EDARADD*, *ELOVL2* and *PDE4C* while Thong’s clock is based on a multiple linear regression (MLR) model using 3 CpGs located in *ELOVL2*, *KLF14* and *PDE4C* (Figure 1). The two Garali’s clocks use 2 to 3 CpGs in *ELOVL2* and are based on MQR or machine learning gradient boosting regressor (GBR, Figure 1). The description of the epigenetic clocks including the regression equations are given in Supplementary Table 1.

### Statistical analysis

All statistical analyzes were performed using RStudio and GraphPad Prism. Correlation analyzes were performed by calculating Pearson’s R coefficient. Differences between DNAmage and chronological age (DNAmage - chronological age) were calculated for each subject, where a negative or positive value indicates an epigenecic age younger or older than the chronological age, respectively. The mean differences of DNAmages and chonological ages between each group and subgroup were assessed using two-tailed Mann-Whitney U tests and the significance threshold was fixed at 0.05.

## RESULTS

### Epigenetic clocks selection and correlation of DNA methylation to chronological ages

In the present study we aimed to estimate the epigenetic age of long-lived individuals as well as their offspring using epigenetic clocks based a small number of CpG sites (Figure 1). We selected four clocks, known as Thong, Bekaert, Garali MQR and Garali GBR clocks, as they have shown a strong correlation and little age differences between DNAmage and chronological age when applied to DNA samples from individuals aged from 0 to 91 years in their original training and testing sets as well as in independent validation sets [17, 18, 35, 36]. These models also presented no or very slight bias according to the sex and to the chronological age [17, 18, 35, 36].

**Figure 2:**
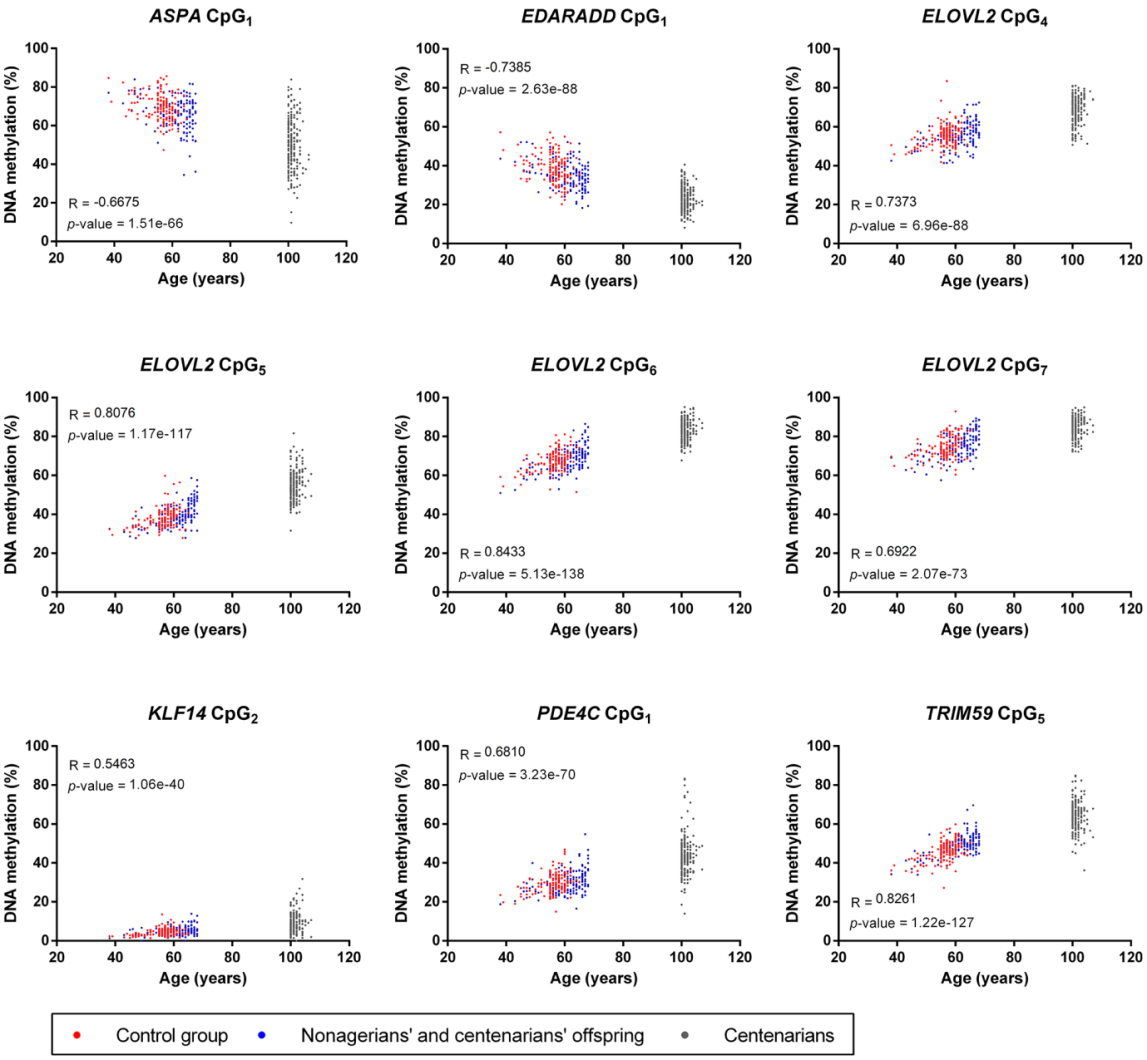
**Correlation analysis between the chronological age and DNA methylation from the nine CpGs included in the four epigenetic clocks used in our study.** For each CpGs, Pearson R coefficients and *p*-values are indicated on the graphs.

Correlation analysis of DNA methylation from the nine CpGs included in the epigenetic clocks and the chronological age of all individuals included in our study showed strong significant correlations for all loci (1.06e-40 ≤ *p*-value ≤ 5.13e-138, Figure 2). *ELOVL2* CpG_7_, *KLF14* CpG_2_ and *PDE4C* CpG_1_ showed moderate positive correlations (0.5463 ≤ R ≤ 0.6922), while *ELOVL2* CpG_4-6_, and TRIM59 CpG_5_ presented strong positive correlations (0.7373 ≤ R ≤ 0.8433, Figure 2). Inversely, *ASPA* CpG_1_ and *EDARADD* CpG_1_ showed a moderate (R=-0.6675) and strong (R=-0.7385) negative correlation, respectively (Figure 2). Of note, some dispersion of DNA methylation values was observed for centenarians at *ASPA* CpG_1_, *KLF14* CpG_2_, *PDE4C* CpG_1_ and *TRIM59* CpG_5_ but not at *EDARRAD* CpG_1_ and *ELOVL2* CpG_4-7_, suggesting that some DNA methylation-based epigenetic clock biomarkers of aging are affected by the epigenetic drift while others are not (Figure 2).

### Centenarians and their offspring present younger DNAmages than their chronological ages

**Figure 3:**
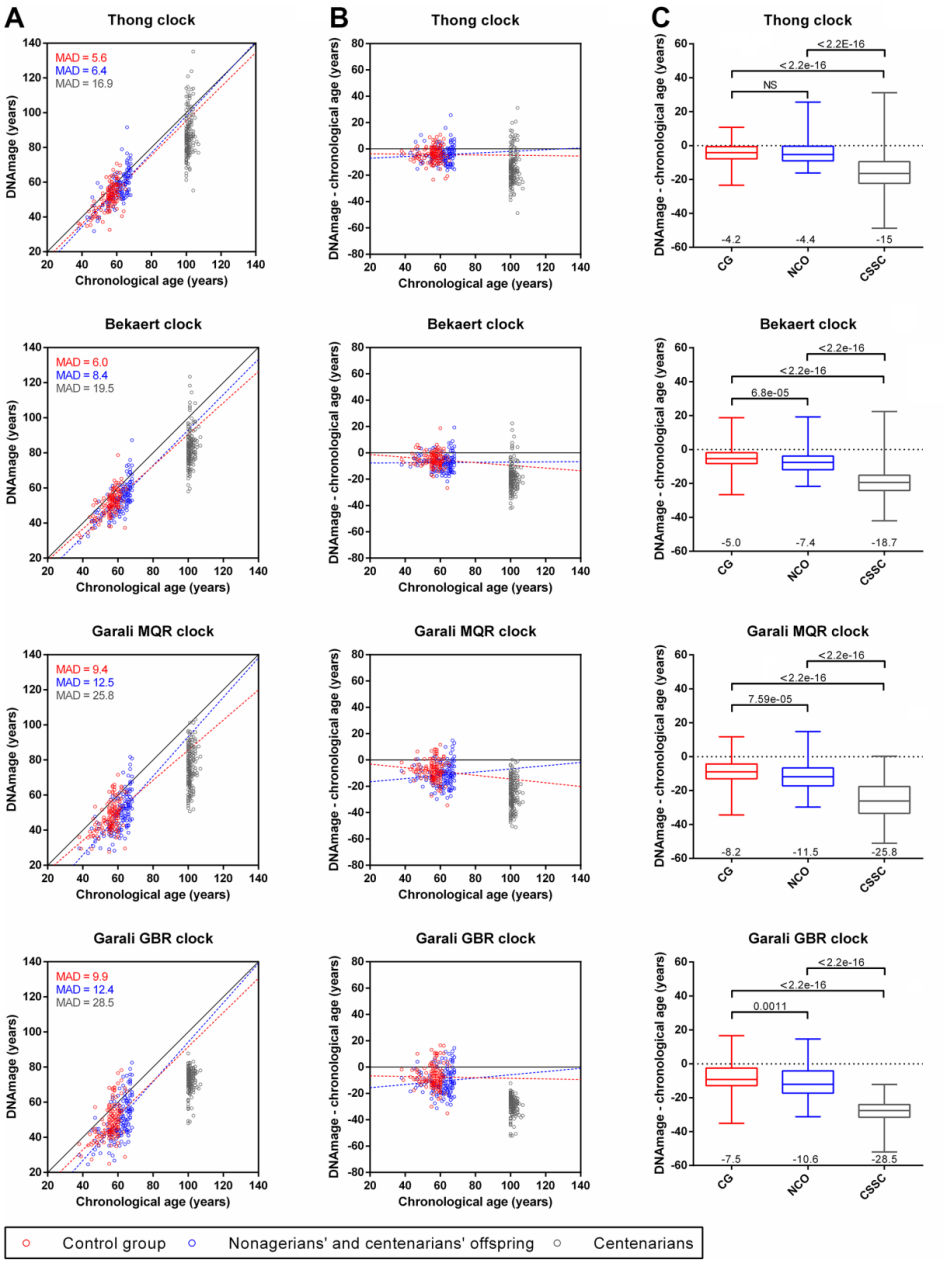
**Comparison of DNAmage and chronological age obtained with the four epigenetic clocks for individuals from the control group (CG), nonagenarian’s and centenarians’ offspring (NCO) and centenarians (CSSC).** (**A**) Scatterplots of the DNAmage and chronological age. The mean absolute deviation (MAD) of DNAmage from chronological age is given for each group. (**B**) Age differences between DNAmage and chronological age plotted against chronological age. Linear regression curves from CG and NCO samples are drawn in red and blue dotted lines (**A**, **B**), respectively. (**C**) Boxplots of DNAmage and chronological age differences according to each group. The mean age difference value is indicated at the bottom of each boxplot, while the *p*-values of the Mann-Whitney U tests are indicated at the top.

We estimated the DNAmage of three groups of individuals including a control group (CG) of individuals from SU.VI.MAX and EFS cohorts from the French general population aged from 38 to 65 years, a group of nonagenarians’ and centenarians’ offspring (NCO) aged from 38 to 68 years and a group of centenarians and semi-supercentenarians (CSSC) from 100 to 107 years (Table 1, Figure 1). DNAmages obtained with the four epigenetic clocks showed quite close values to chronological ages from individuals from the control group (5.6 ≤ MAD ≤ 9.9) and NCO group (8.4 ≤ MAD ≤ 12.5), with greater differences in single-locus (Garali MQR and GBR) than multi-loci (Thong and Bekaert) epigenetic clocks (Figure 3A). Linear regression curves obtained for CG and NCO groups were slightly below but almost parallel to the x=y line, indicating a slight underestimation of DNAmage compared to chronological age as well as a low bias for the different age ranges with the four epigenetic clocks (Figure 3A, 3B). In contrast, CSSC presented DNAmages highly different from their chronological age (16.9 ≤ MAD ≤ 28.5), which were mainly younger than their chronological ages and under both the NCO and CG regression lines (Figure 3A, 3B).

When comparing DNAmage and chronological age differences, a younger DNAmage was observed for CG individuals (6.2 years on average, mean of the four epigenetic clocks) as well as for NCO (8.5 years on average), respectively, while this difference was strongly accentuated in CSSC (22 years on average, Figure 3B, 3C). Thus, compared to CG individuals, NCO presented slightly younger DNAmage (2.3 years on average, corresponding to “DNAmage – chronological age” value of NCO minus that of CG) and these age differences were statistically significant in three of four clocks (Bekaert, Garali MQR and GBR clocks, Figure 3C). Moreover, CSSC also presented strongly younger DNAmages compared to CG individuals (15.8 years on average) and NCO (13.5 years on average), where the differences between DNAmage and chronological age among CSSC were significantly different from the CG and NCO (*p*-values < 2.2e-16, Figure 3C). As the three groups presented different sex ratios, we further evaluated if the observed DNAmage differences between groups could be a consequence of this sex bias. Our results showed no statistically significant differences between male and female inside each group no matter which epigenetic clock was used, with one exception with Bekaert clock for CG subjects (*p*-value=0.0123, Supplementary Figure 2).

Similarly, we also investigated whether some characteristics in the NCO and CSSC could also impact the differences observed between DNAmage and chronological age. In NCO, grouping individuals according to the age of the oldest parent i.e. nonagenarian’s vs centenarians, or to the sex of the oldest parent (NC mother vs NC father) did not showed clear evidence of DNAmage differences, despite of a slightly significant *p*-value (0.0460) for the first comparison with Bekaert clock (Supplementary Figure 3A, 3B). In CSSC, distinguishing centenarians from semi-supercentarians, or considering them according their time to death after collection of their blood samples did not reveal evidence of DNAmage difference, except for the last comparison presenting a slightly significant *p*-value (0.0396) with Garali MQR clock (Supplementary Figure 3C, 3D).

Taken together, our results showed that French CSSC and NCO presented younger DNAmage than CG individuals, suggesting that epigenetic aging and potentially biological aging is slowed down in long-lived individuals as well as their offspring.

## DISCUSSION

The present study aimed to assess DNAmage in French long-lived individuals and their offspring and also aimed to evaluate whether this measure of epigenetic aging could be affected compared to individuals from the French general population. Thus, four previously published epigenetic clocks based on 2 to 4 CpGs in 1 to 4 genes (Figure 1) were selected and applied to blood DNA samples from 506 individuals, including 149 individuals of the general population aged from 38 to 65 years, 143 nonagenarians’ and centenarians’ offspring aged from 38 to 68 years and 214 centenarians and semi-supercentenarians aged from 100 to 107 years (Table 1). To our knowledge, this is the first time that DNAmage was investigated in such a large cohort of centenarians and of long-lived individuals’ offspring using epigenetic clocks based on a small number of CpG sites [30–32].

The DNA methylation analysis of the nine CpGs used in the four epigenetic clocks showed moderate to strong positive and negative correlations for all CpGs (R < -0.66 or > 0.54, Figure 2), which was expected and consistent with the results obtained in the original or validation studies developing these epigenetic clocks [17, 18, 35, 36]. An interesting result was the increase in the dispersion of DNA methylation of 4 CpG sites (*ASPA* CpG_1_, *KLF14* CpG_2_, *PDE4C* CpG_1_ and TRIM59 CpG_5_) in CSSC compared to NCO and CG individuals that was not or less visible for the five others (*EDARRAD* CpG_1_ and *ELOVL2* CpG_4-7_, Figure 2). This divergence could be considered as a result of the epigenetic drift, which is characterized by the progressive divergence of DNA methylation of CpG sites occurring during aging either stochastically or under the influence of environment [6, 7, 10]. Although epigenetic clocks and epigenetic drifts are frequently opposed and considered as two distinct mechanisms [6, 7], our results showed that both phenomena could coexist inside a same CpG, including some of the best epigenetic clock biomarkers of aging (Figure 2). This epigenetic drift was not visible for these CpGs in other studies as it might only appear in long-lived individuals of extreme ages [13, 17, 35, 41]. In contrast, despite their extreme age, DNA methylation of the other CpGs sites analyzed remained tighten in CSSC, suggesting that some epigenetic clock biomarkers of aging might be completely resistant or insensitive to the epigenetic drift.

DNAmage analysis of individuals from CG, NCO and CSSC using the four epigenetic clocks showed a global under-evaluation of DNAmage for all groups compared to their chronological age, less in the CG individuals (6.2 years) and NCO (8.5 years) and more in CSSC (22 years, Figure 3). These results were surprising for the CG individuals, as no deviation of DNAmage from their chronological age was expected in individuals from the general population, as described in previous studies performed in our lab with the same epigenetic clocks [17, 18]. However, under- and over-estimation of the DNAmage relative to chronological age in individuals from the general population were common with several epigenetic clocks, both with those based on small number of CpGs using high resolution technologies [17, 42] and those based on a large number of CpGs using epigenotyping microarrays [30, 43]. This was mainly attributed to technical variations for absolute quantification of DNA methylation for both type of epigenetic clocks as well as the bio-informatic algorithms used for data processing and normalization for the second types of epigenetic clocks [17, 30, 42, 43]. In the CG, 21 EFS DNA samples were previously analyzed with the four epigenetics clocks in two former studies [17, 18], and the comparison of both DNAmage from the same samples showed a lower value in our current study with each clock (1.3, 3.1, 3.3 and 2 years for Thong, Bekaert, Garali MQR and GBR, respectively, data not shown). Thus, some technical variations might have led to the under-evaluation of DNAmage from CG individuals, whose inclusion in our study was absolutely necessary for DNAmage comparisons.

Compared to their chronological age, DNAmage of CSSC was strongly underestimated (15 to 28.5 years in average), which was still strongly significantly underestimated when compared to CG DNAmage (10.8 to 21 years in average, *p*-values < 2.2e-16, Figure 2). This might indicate that the epigenetic clock and potentially aging were decelerated in exceptionally long-lived individuals, who presented younger DNAmage and potentially also younger biological age. This idea is reinforced as DNAmage and epigenetic clocks were considered as a better molecular predicator of biological age than chronological age, which could sometimes also predict lifespan and healthspan [11, 19, 20, 44]. Our results were consistent with three other studies that also investigated DNAmage in long-lived individuals, including nonagenarians, centenarians and/or semi-supercentenarians from Italian, Israeli and Australian populations, in which long-lived individuals presented younger DNAmage than chronological age [30–32]. Similarly, younger DNAmage (from 0.2 to 3.3 years in average) were also highlighted in NCO compared to CG individuals with the four epigenetic clocks, three of which showed significant differences (Figure 3C). This observation was also found in two of the three previously mentioned studies that investigated DNAmage in LLI offspring [31, 32], suggesting that epigenetic and biological aging could also be decelerated in these individuals. It should be noted that the differences observed between each group when comparing DNAmage and chronological age could also have been influenced by specific exposures of each cohort and/or mortality selection, notably as the three groups presented different mean chronological ages, even between individuals from CG and NCO (56 vs 61.2 years, Table 1). Compared to our study, the three aforementioned studies were performed on a lower number of participants in the three groups of LLI (23, 24 and 75 subjects) and the two groups of NCO (18 and 63 subjects) and relied on epigenetic clocks based on a large number of CpG sites (71 to 513 CpGs) using epigenotyping microarrays technologies [30–32]. Thus, our results showed that the use of epigenetic clocks based on a very small number of loci (from 1 to 4) and of CpG sites (from 2 to 4) could be sufficient to reveal an alteration of the global epigenetic clock of individuals due to a particular health condition, i.e. extreme longevity in our study. This could potentially be explained by the loci used in our study, which were selected among the very best DNA methylation-based epigenetic clock biomarkers of aging [7, 13, 17, 35, 41, 45] and which could also potentially be among the most sensitive to alteration of the global epigenetic clock due to health conditions and/or environment. Other studies investigating DNAmage using epigenetic clocks based on few CpG sites in individuals with particular health conditions, including elite athletes [24], chronic lymphocytic leukemia [46], Alzheimer and Graves’ disease [47] also revealed significant differences when compared to a control groups. Thus, these results and ours indicate that epigenetic clocks based on a small number of CpGs could be highly informative and used in place of more complex epigenetic clocks based on a large number of CpGs for a rapid and high-throughput analysis of DNAmage at lower costs. However, these epigenetic clocks would not allow more sophisticated analyses that are included in some epigenotyping microarray-based epigenetic clocks [16, 20, 26].

Regarding the sex of all subjects analyzed in our study, we did not have the possibility to balance the sex ratio between each group due to sample availability constraints. However, comparisons between men and women subjects did not show any significant differences between them, except for one comparison among the twelve performed (Supplementary Figure 2). While some studies using epigenetic clocks based on a large number of CpGs (Horvath and Hannum clocks) showed that women had slightly younger DNAmage than men, which could be expected due to their different life expectancy [48, 49], others using epigenetic clocks based on a small number of CpGs, including some of ours, did not show such a difference [17, 35]. This indicates that the epigenetic clocks used in our study cannot detect sex-related DNAmage differences, which could potentially be due to an insufficient number of CpGs and/or the absence of certain specific CpGs in these clocks. However, they should be suitable for comparing groups of individuals with different sex ratios without generating any sex bias. Finally, when we compared the DNAmage in CSSC according to their age or their time to death and NCO according to the sex or the age of the oldest parents, we found no consistent differences among the four epigenetic clocks, suggesting that these parameters might not differentially affect – or only slightly – DNAmage. However, some studies relying on Horvath and Hannum clocks have showed that DNAmage could predict all-cause mortality [23] and time to death [50] in cohorts of individuals with an average age of 57 to 77.1 years. In our study, the epigenetic clocks used were unable to detect differences in DNAmage of centenarians with variable time to death after collection. Although these results could be due to the small differences in time to death of the two groups of CSSC – most CSSC (86%) died within 4 years after collection –, it should also be kept in mind that these epigenetic clocks could potentially be less sensitive for detecting slight differences in epigenetic and biological aging of individuals than those based on a large number of CpGs and should be used accordingly.

## CONCLUSIONS

We investigated for the first time the DNAmage of three groups of French subjects including CSSC, NCO and a control group from the general population using four epigenetic clocks based on a small number of CpG sites. DNA methylation analysis of the nine CpG included in the epigenetic clocks showed that epigenetic drift was sometimes only visible in extremely old individuals. Epigenetic clock analysis showed that NCO and CSSC presented DNAmages slightly and strongly younger than their chronological ages compared with CG individuals, respectively. This suggests a decelerated epigenetic and biological aging in these two groups of individuals, confirming the results of three other studies performed on Italian, Australian and Israeli long-lived individuals. In addition, our study also demonstrated the possibility of using epigenetic clocks based on a small number of CpG sites to reveal DNAmage and chronological age differences between individuals with different life expectancy.

## Supplementary Material

Supplementary Figures

Supplementary Table 1
