# Supplementary material for: Centenarians consistently present a younger epigenetic age than their chronological age with four epigenetic clocks based on a small number of CpG sites
Source: Aging (Albany NY). 2022 Oct 3;14(19):7718–33. doi: 10.18632/aging.204316 (PMC9596211; doi:10.18632/aging.204316)
Supplement: Supplementary Figures [file aging-14-204316-s001.pdf]

SUPPLEMENTARY FIGURES

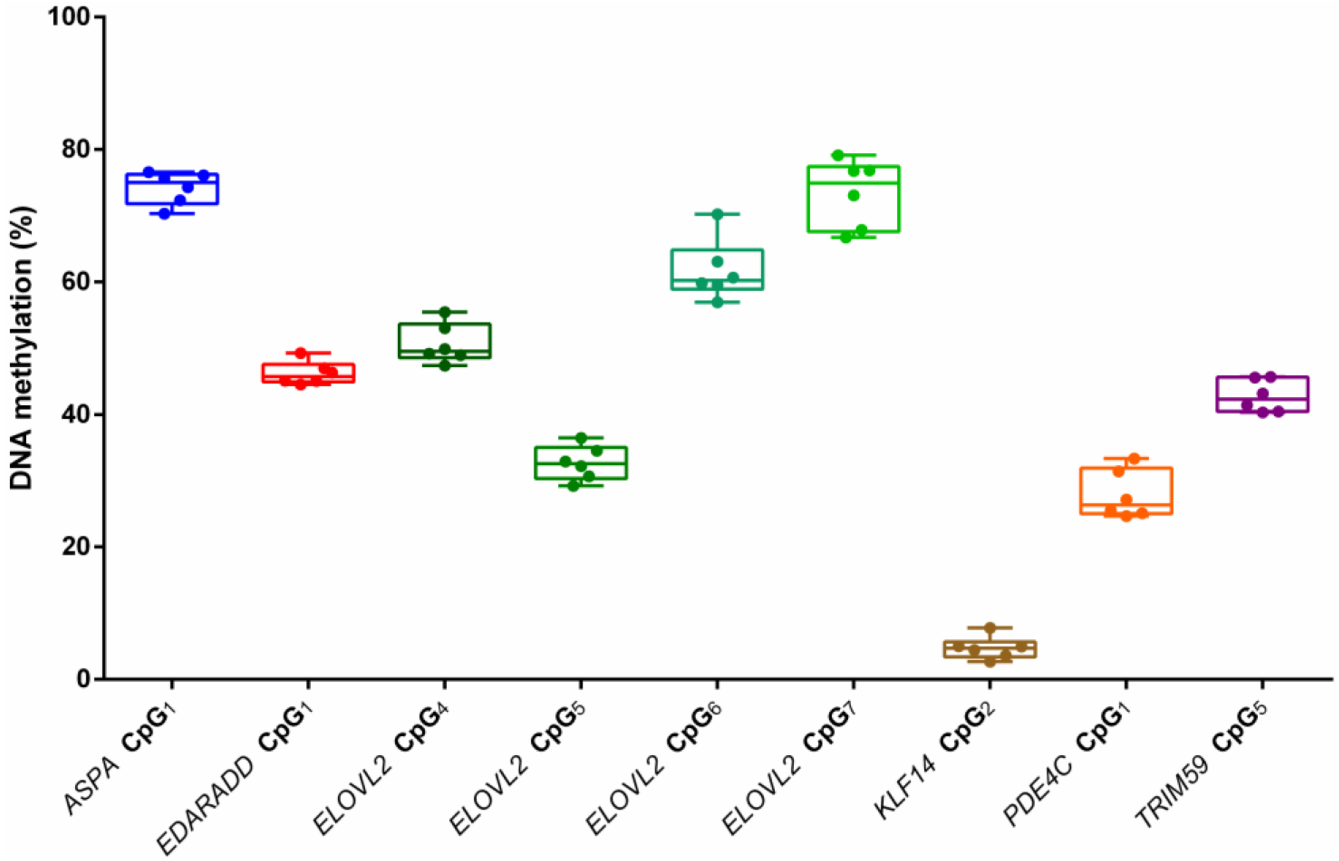

Supplementary Figure 1. DNA methylation of the nine CpGs used in our study obtained from a replicate commercial whole blood DNA sample (Promega) present on every bisulfite-treated PCR plate. A total of six 96-well PCR plates was used for bisulfite conversion in our study.

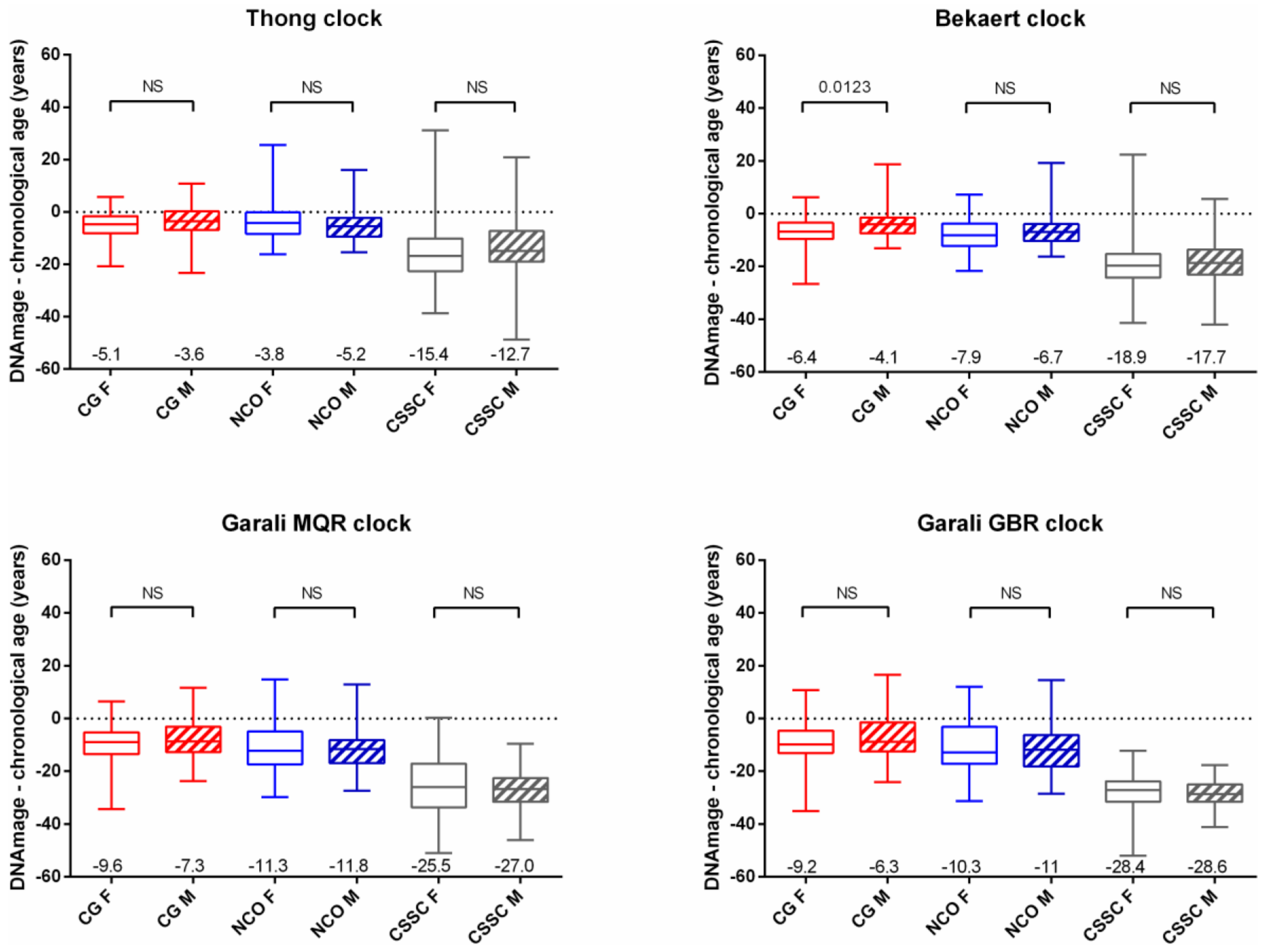

Supplementary Figure 2. Boxplots of DNAmage and chronological age differences according to sex (clear box plots, F = women; hatched box plots, M = men) in control group of disease-free individuals (CG), nonagenarians and centenarians' offspring (NCO) and centenarians and semi-supercentenarians (CSSC). The mean age difference value is indicated at the bottom of each boxplot, while the p-values of the Mann-Whitney U tests are indicated at the top.

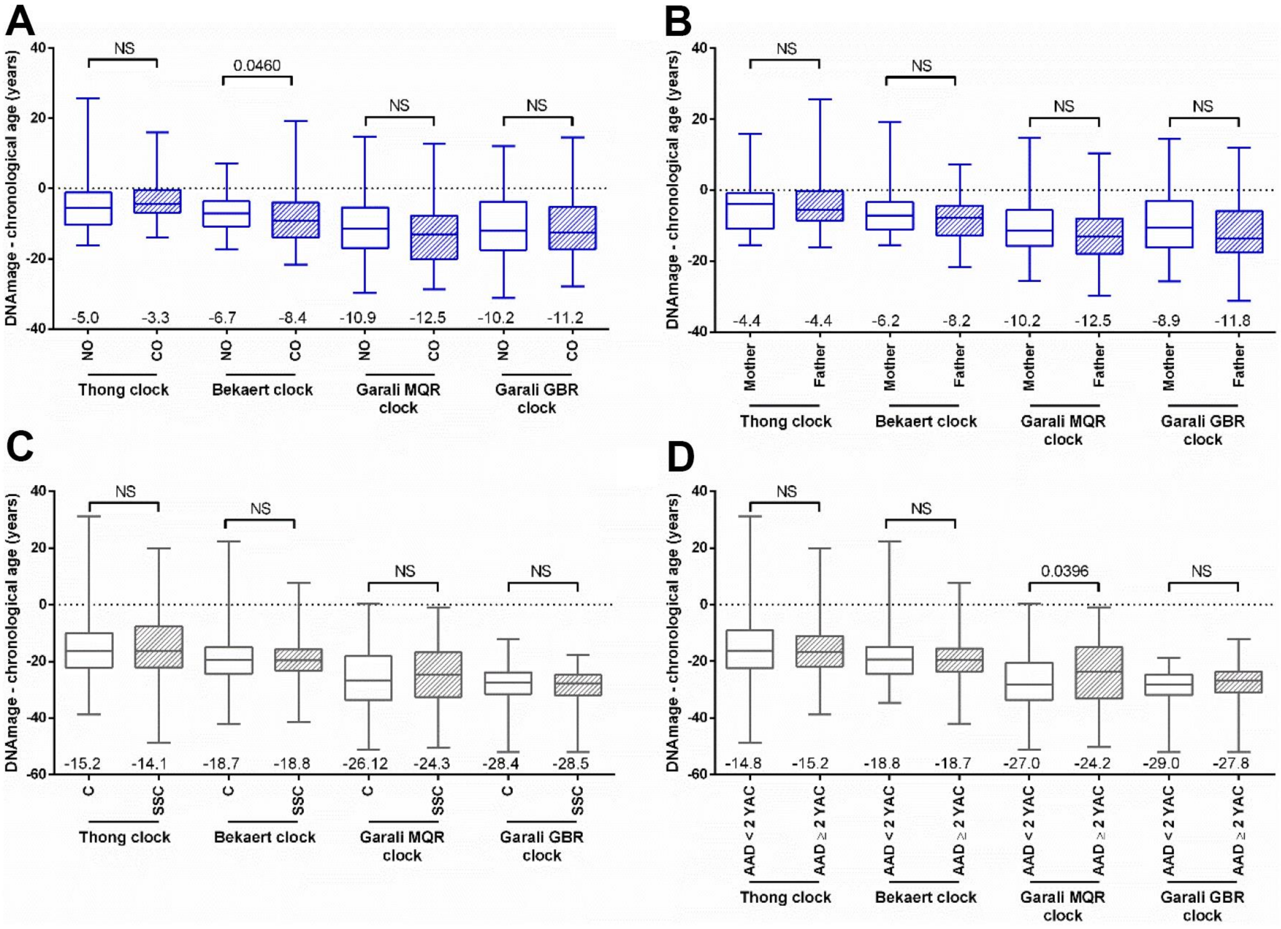

Supplementary Figure 3. Comparison of DNAmage and chronological age differences obtained for sub-groups inside CSSC and NCO of the CEPH aging cohort. (**A**) DNAmage and chronological age differences between nonagenarians' offspring (NO, 90 years < age at death of the oldest parent < 99 years, n = 90) and centenarians' offspring (CO, age at death of the oldest parent ≥ 99 years, n = 53). (**B**) DNAmage and chronological age differences between offspring from a nonagenarians'/centenarians' mother (Mother, n = 60) and offspring from a nonagenarians'/centenarians' father (Father, n = 83). (**C**) DNAmage and chronological age differences between centenarians (C, n = 170) and semi-supercentenarians (SSC, n = 44). (**D**) DNAmage and chronological age differences between CSSC with and age at death below 2 years after collection (AAD < 2 YAC, n = 119) and CSSC with and age at death of at least 2 years after collection (AAD ≥ 2 YAC, n = 95). The mean age difference value is indicated at the bottom of each boxplot, while the p-values of the Mann-Whitney U tests are indicated at the top.
