# Supplementary material for: Centenarians consistently present a younger epigenetic age than their chronological age with four epigenetic clocks based on a small number of CpG sites
Source: Aging (Albany NY). 2022 Oct 3;14(19):7718–33. doi: 10.18632/aging.204316 (PMC9596211; doi:10.18632/aging.204316)
Supplement: Supplementary Table 1 [file aging-14-204316-s002.pdf]

SUPPLEMENTARY TABLE

Supplementary Table 1. Description of the four epigenetic clocks used.

Epigenetic clock	Mathematical approach	Formula
Bekaert [1]	Multiple Quadratic Regression (MQR)	26.444119 - 0.201902 x *ASPA* (CpG_1) - 0.239205 x *EDARADD* (CpG_1) + 0.0063745 x *ELOVL2* (CpG_6)^2 + 0.6352654 x *PDE4C* (CpG_1)
Thong [2]	Multiple Linear Regression (MLR)	-20.372 + 0.830 × *ELOVL2* (CpG_5) + 1.723 × *KLF14* (CpG_2) + 0.715 × *TRIM59* (CpG_5)
Garali [3]	Multiple Quadratic Regression (MQR)	13.4944951 - 0.8224263 x *ELOVL2* (CpG_6) - 0.0001978 x *ELOVL2* (CpG_4)^2 + 0.0143482 x *ELOVL2* (CpG_6)^2 + 0.004438 x *ELOVL2* (CpG_7)
Garali [3]	Gradient Boosting Regressor (GBR)	DNA methylation of *ELOVL2* (CpG_6 and CpG_7) was used as an input testing set in the provided GBR R code using a training set of 1028 samples [3]

REFERENCES

1. Bekaert B, Kamalandua A, Zapico SC, Van de Voorde W, Decorte R. Improved age determination of blood and teeth samples using a selected set of DNA methylation markers. Epigenetics. 2015; 10:922–30. https://doi.org/10.1080/15592294.2015.1080413 PMID:26280308

2. Thong Z, Chan XLS, Tan JYY, Loo ES, Syn CKC. Evaluation of DNA methylation-based age prediction on blood. Forensic Science International: Genetics Supplement Series. 2017; 6:e249–51. https://doi.org/10.1016/j.fsigss.2017.09.095

3. Garali I, Sahbatou M, Daunay A, Baudrin LG, Renault V, Bouyacoub Y, Deleuze JF, How-Kit A. Improvements and inter-laboratory implementation and optimization of blood-based single-locus age prediction models using DNA methylation of the ELOVL2 promoter. Sci Rep. 2020; 10:15652. https://doi.org/10.1038/s41598-020-72567-6 PMID:32973211
